# Blind Manipulation of Deformable Objects Based on Force Sensing and Finite Element Modeling

**DOI:** 10.3389/frobt.2020.00073

**Published:** 2020-06-09

**Authors:** Jose Sanchez, Kamal Mohy El Dine, Juan Antonio Corrales, Belhassen-Chedli Bouzgarrou, Youcef Mezouar

**Affiliations:** Université Clermont Auvergne, CNRS, SIGMA Clermont, Institut Pascal, Clermont-Ferrand, France

**Keywords:** deformation servoing, finite-element modeling, tactile sensing, robotic manipulation, force sensing

## Abstract

In this paper, we present a novel pipeline to simultaneously estimate and manipulate the deformation of an object using only force sensing and an FEM model. The pipeline is composed of a sensor model, a deformation model and a pose controller. The sensor model computes the contact forces that are used as input to the deformation model which updates the volumetric mesh of a manipulated object. The controller then deforms the object such that a given pose on the mesh reaches a desired pose. The proposed approach is thoroughly evaluated in real experiments using a robot manipulator and a force-torque sensor to show its accuracy in estimating and manipulating deformations without the use of vision sensors.

## 1. Introduction

As deformable objects are ubiquitous in many industries, automating their manipulation would have a great social impact. For instance, robots could perform tasks that are either dangerous or monotonous for workers. Examples of manipulation of deformable objects can be found in the automobile and aerospace industries, where cables and wires must be connected in order to assemble motors; in health care, where clothes are handled to dress disabled people; and in the food industry where meat and produce have to be processed with care. This has sparked interesting robotic solutions that attempt to address issues such as automating the manufacture of motors by manipulating cables as proposed by Rambow et al. (2012), Roussel and Ta (2015), and Shah and Shah (2016), using robots to perform clothing assistance as described by Yu et al. (2017) and Erickson et al. (2017). and even food handling as proposed by Bac et al. (2017) and Lehnert et al. (2017). The interest in the robotic community to address the sensing and manipulation of deformable objects has been increasing in recent years as shown by the number of works covered in different surveys as presented by Henrich and Wörn (2000), Khalil and Payeur (2010), and Sanchez et al. (2018a).

**Figure 1:**
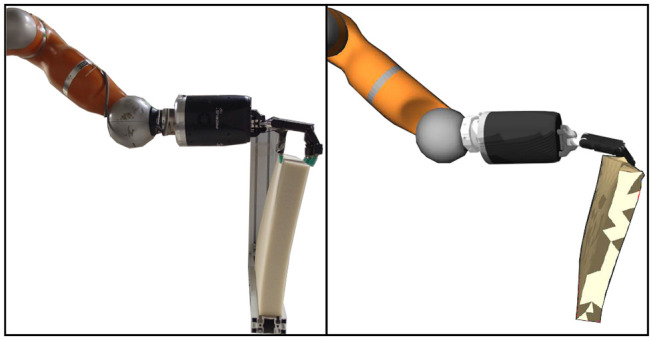
Real deformation of a block-like object, shown on the left side, compared to the estimation of the proposed approach, visualized in RViz, shown on the right side.

In this paper we propose an approach to both sense and manipulate the deformation of soft object^1^. Specifically, we extend our previous work where a pipeline to estimate the deformation of a soft object was proposed in Sanchez et al. (2018b). This deformation sensing pipeline requires an input force and an initial mesh to output the updated mesh that describes the deformation of the object. Here, in addition to enhancing the sensing capabilities of our previous work, we develop a controller to manipulate a deformable object. The sensing improvements are mainly due to changing the input sensor from a set of tactile sensors on the finger tips of a robotic hand to a single force-torque sensing attached to the end of a robotic arm. Regarding the manipulation of a soft object, replacing a robot hand by a robotic manipulator simplifies the manipulation problem since a robotic hand is limited to its finger motions (e.g., opening and closing the hand); while a seven degree of freedom manipulator is able to move in the 3D Cartesian space. In order to perform this manipulation we simplify the problem by controlling the position of a single pose on the mesh. An comparison between our estimation and a real world deformation can be seen in Figure 1.

Following this introduction we review the state of the art for robotic sensing and manipulation of deformable objects in section 2. In section 3, we detailed our approach to estimate the deformation of an object while being manipulated as well as controlling its deformation. An experimental validation of the proposed approach is presented in section 4 followed by a discussion of the experimental results in section 5. Finally, our concluding remarks and future work are summarized in section 6.

## 2. Related Work

Two decades ago, one of the first works focusing on controlling the shape of a deformable object was presented by Wada et al. (1998), where the problem was formulated as an Indirect Simultaneous Positioning (ISP) problem. ISP consists in defining two sets of points, namely, controlled and manipulation points. Where the former are points on the surface of the object (usually away from the edges such that are not graspable), while the latter are defined by positions where a robotic manipulator is grasping the object.

Following the work of Wada, other researchers have tackled the ISP problem using vision to obtain the position of the controlled points. Navarro-Alarcón et al. in a series of works, proposed a way to control the configuration of a deformable object using visual servoing. Here, the configuration of the object is described using *deformation feature* vectors, based on a set of points tracked using markers. The proposed deformation vectors were defined by a set of points in an object to control a point, a distance or angle between two points, and a curvature described by three points. These approaches rely on a deformation Jacobian, which here refers to a matrix mapping the motion of the grippers to the deformation of the object. In Navarro-Alarcon et al. (2013), the deformation Jacobian is estimated using the Broyden method, which computes the Jacobian once at the beginning and then approximates it at each iteration using the previous Jacobian and the changes of the feature vectors and the end-effector's pose; and, in Navarro-Alarcon et al. (2014), they proposed a new estimation that used views from multiple cameras. However, both of these approaches were limited as they control the deformation features on a plane, namely in the image space. This was later addressed in Navarro-Alarcon et al. (2016), by using stereo-vision to track the points in 3D and subsequently define the deformation feature vectors also in 3D.

A similar approach proposed by Alambeigi et al. (2018) extended its application to heterogeneous objects while being robust to disturbances, e.g., the objects were filled with water beads (heterogenous) and then cut (disturbance) while the controller was running. To achieve this, instead of relying only on a deformation Jacobian, they combined it with an image Jacobian to consider both the deformation behavior of the object as well as the feedback points obtained by the vision system. Recently, Hu et al. (2018), using an RGB-D camera, were able to obtain a much faster convergence in the deformation control than the approaches proposed in Navarro-Alarcon et al. (2014, 2016) by using Gaussian Process Regression (GPR) to estimate the deformation Jacobian, since GPR is able to model non-linearities.

Instead of relying on vision, Berenson (2013) used simulated objects described by a set of points, where the positions were assumed to be known at any time. Unlike the approaches described above, Berenson estimated the deformation Jacobian by introducing the concept of *diminishing rigidity* which assumes that the controlled points closer to manipulated points (e.g., a point where the object is being held) behave rigidly and the farther the controlled points are from manipulated points the less rigid they become.

A novel approach for controlling the shape of an object without requiring an ISP formulation, was recently proposed in Navarro-Alarcon and Liu (2018), where the object's contour was described by Fourier coefficients. Thus, instead of controlling the deformation of an object based on a set of points, they deformed the object such that the contour of the object was similar to the desired one.

Other works have focused on sensing the deformation of an object rather than controlling its shape using vision, either by relying on a mesh model as proposed in Petit et al. (2015), Fugl et al. (2012), Schulman et al. (2013), and Frank et al. (2010) or being model-free (see Cretu et al., 2012; Güler et al., 2015). Due to space limitations we do not review the works focused on deformation sensing, but we refer the interested reader to a recent survey were these works are thoroughly reviewed (Sanchez et al., 2018a).

## 3. Approach

As described in the previous section, most work concerning deformation control has either relied on vision sensing or assumed that the object's shape (e.g., a set of points describing the object) is known at any time. However, vision systems are sensitive to variations in illumination, have difficulties segmenting objects with similar colors to their backgrounds and, perhaps most importantly, they are limited by occlusions arising while manipulating objects. In order to provide a complementary sensing modality, we propose a method using force sensing, coupled with a deformation model, to estimate the shape of an object while being deformed.

We propose a manipulation controller, shown in **Figure 3**, which uses an improved version of the deformation sensing pipeline proposed in Sanchez et al. (2018b) and a novel force sensor model, described in Mohy el Dine et al. (2018), that estimates the pure contact forces generated while deforming an object grasped by a robot manipulator. This approach shows a potential application when vision is compromised, e.g., when a room is too dark or cluttered.

### 3.1. Force Sensor Model

**Figure 2:**
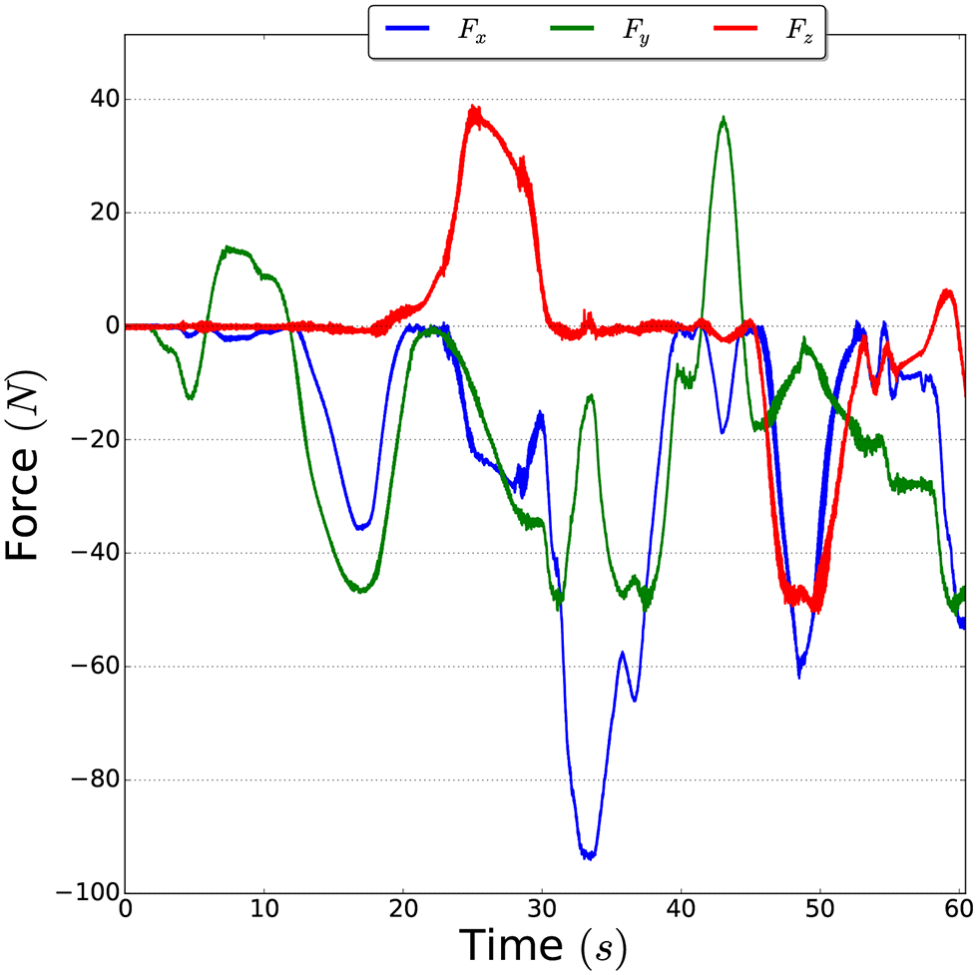
Example of non-contact forces measured by the force-torque sensor when the robot arm is moved without any contacts.

Since the input to the deformation sensing pipeline is the contact force that deforms the object, we obtain this force using a force-torque sensor. However, as force-torque sensors not only measure contact forces but also non-contact forces generated by gravity, inertia, Coriolis and centrifugal forces (as depicted in Figure 2); it is necessary to first estimate the non-contact forces to then subtract them from the measured forces in order to obtain the pure contact forces. Thus, the output of a force-torque sensor can be expressed as:

(1) $$\begin{array}{l} {\text{F}}_{meas}={\text{F}}_{nc}+{\text{F}}_{c} \end{array}$$

where **F**_*meas*_ is the measured force, i.e., the output of a force-torque sensor, **F**_*c*_ represents the contact forces and the non-contact forces **F**_*nc*_ are defined as:

(2) $$\begin{array}{l} {\text{F}}_{nc}={\text{F}}_{gravity}+{\text{F}}_{inertia}+{\text{F}}_{coriolis}+{\text{F}}_{centrifugal} \end{array}$$

In order to estimate these non-contact forces from a force-torque sensor, we use a recently proposed observer that was shown to outperform analytical based methods by using a Recurrent Neural Network. The Recurrent Neural Network observer (RNNOB) proposed in Mohy el Dine et al. (2018), takes as input the orientation and twist of the robot end-effector frame w.r.t. the robot base frame [**o**^*ee*^, **υ**^*ee*^, **ω**^*ee*^]^*T*^ which are obtained by the first and second order forward kinematics; as well as the linear acceleration **α**^*IMU*^ published by an IMU sensor. The output of the RNNOB is the estimated non-contact force w.r.t. the sensor frame ${\hat{\text{F}}}_{nc}^{s}$. Then, the pure contact forces, expressed in the sensor frame, can be estimated as:

(3) $$\begin{array}{l} {\text{F}}_{c}^{s}={\text{F}}_{meas}^{s}-{\hat{\text{F}}}_{nc}^{s} \end{array}$$

The network's architecture consisted of two hidden layers, with 15 and 10 LSTM units, and it was trained over 50 epochs using 80% of the data set (~10,000 data points). The sequence length of the input layer was of 100 time steps (0.2 s). In the output layer, we applied Stochastic Gradient Descent with a learning rate of 0.01 to minimize the mean square error of the regression problem. A hyperbolic tangent sigmoid function was used as the activation function between the layers.

### 3.2. Deformation Sensing Pipeline

To estimate the deformation of the object we improve the sensing pipeline proposed in our previous work (Sanchez et al., 2018b). Namely, we replaced the tactile sensors by a single force-torque sensor and, instead of pushing the objects with the fingertips of a robot hand, the object is grasped by a robot hand. The advantages of grasping an object over pushing it with the fingertips, are twofold:

1. The object can be manipulated in multiple directions (i.e., it can be moved up/down and side to side and be pulled and pushed).
2. The contact locations can be assumed to remain fixed.

**Figure 3:**
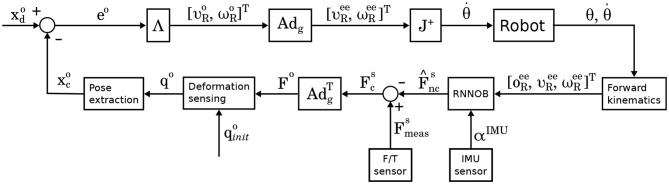
Block diagram of the proposed deformation controller. The current and desired poses, ${\text{x}}_{c}^{o}$ and ${\text{x}}_{d}^{o}$, respectively, are described w.r.t. the object frame. As the robot expects the end-effector twist expressed in the robot base frame {*R*}, the twist expressed on the object frame, namely $\left[\upsilon_{R}^{o},\omega_{R}^{o}\right]$, must be multiplied by an adjoint matrix **Ad**_*g*_ relating these two frames in order to obtain the desired twist ($\left[\upsilon_{R}^{ee},\omega_{R}^{ee}\right]$).

The first advantage allows us to better control the object, where as the second advantage improves the estimation since the location of the contact forces is known and constant (e.g., no slippage of contact). As shown in Figure 3, the deformation sensing pipeline takes as input the initial state of a mesh **q**_*init*_ and a force *F* to estimate the mesh deformation. Formally, a mesh **q** ∈ ℝ^3*n*^ is represented as set of *n* nodes connected by tethrahedral elements. The mesh's deformation is estimated by applying the external forces and propagating the internal forces throughout the mesh. By solving the following differential equation we can obtain the updated positions of the nodes:

(4) $$\begin{array}{l} {\text{F}}_{ext}=\text{M}\ddot{\text{q}}+\text{D}\dot{\text{q}}+{\text{F}}_{int}\left(\text{q}\right) \end{array}$$

where ${\text{F}}_{ext}\in {\mathbb{R}}^{3n}$ is a vector of the three-dimensional forces applied to each node *n*. The position, velocity and acceleration of each node is represented by **q**, $\dot{\text{q}}$, and $\ddot{\text{q}}$, respectively, with **q** ∈ ℝ^3*n*^. The mass matrix is **M** ∈ ℝ^3*n*×3*n*^ and **D** represents the damping matrix.

Since the deformation sensing pipeline requires the input force to be described w.r.t. the object frame, we transform the force using the adjoint matrix $Ad_{g}^{T}$, as obtained by the force sensor model described in section 3.1, as follows:

(5) $$\begin{array}{l} {\text{F}}^{o}=Ad_{g}^{T}\cdot {\text{F}}_{c}^{s} \end{array}$$

### 3.3. Manipulating Deformation

**Figure 4:**
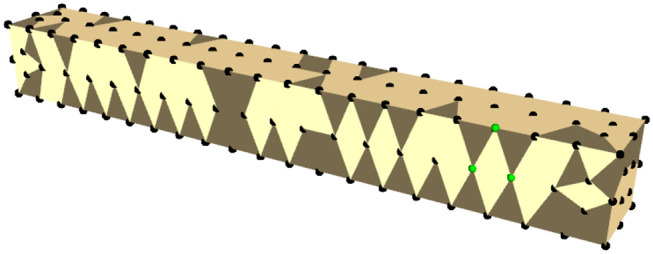
Simulated mesh of a bar-like object. The mesh nodes are shown in black and the nodes used to extract a pose are in green.

Since controlling the complete shape of an object (e.g., all the nodes of a mesh) is a heavily underactuated problem, except for trivial cases, we reduce the control problem by focusing on controlling a single pose on the mesh. To this end, we first must extract a suitable pose from the mesh and consequently control that pose such that it reaches a target pose. To extract a pose we exploit the fact that the nodes of the mesh are connected by tetrahedra so three suitable nodes would form a triangle as shown in Figure 4. By selecting three neighboring nodes (*p*_1_, *p*_2_, *p*_3_) from the mesh, we can extract a pose as follows:

(6) $$\begin{array}{l} \text{c}=\frac{1}{3}\overset{3}{\underset{i=1}{\sum }}{\text{p}}_{i} \end{array}$$

(7) $$\begin{array}{l} \text{n}=\left({\text{p}}_{2}-{\text{p}}_{1}\right)\times \left({\text{p}}_{3}-{\text{p}}_{1}\right) \end{array}$$

(8) $$\begin{array}{l} s=\cos\left(\frac{\pi }{2}\right),\;\text{v}=\text{n}\cdot \frac{\sin\left(\frac{\pi }{2}\right)}{{\left\|\text{n}\right\|}_{2}} \end{array}$$

(9) $$\begin{array}{l} \text{x}={\left[\text{c},\left(s,\text{v}\right)\right]}^{T} \end{array}$$

where **p**_*i*_ is the position of the node at the *i*−th index of the mesh **q** and **n** represents the normal of the plane formed by the points. The position and orientation of the pose are given by the centroid **c** and the quaternion, respectively, where *s* is the scalar part of the quaternion and **v** is the vector part. To manipulate the mesh to a desired pose, let **x**_*c*_ denote the current pose, as extracted by the method described above, and **x**_*d*_ the desired pose for the mesh to reach. Then, we can define an error signal as follows:

(10) $$\begin{array}{l} \text{e}={\text{x}}_{d}-{\text{x}}_{c} \end{array}$$

Subsequently, we can transform the error **e** into a twist command by multiplying it by a diagonal gain matrix **Λ**. As the twist is expressed in Cartesian space, we compute the joint velocities to command a robotic arm by joint velocity control as:

(11) $$\begin{array}{l} \dot{\theta }={\text{J}}^{+}\left(Ad_{g}\cdot \Lambda \cdot \text{e}\right) \end{array}$$

where **J**^+^ is the Moore–Penrose inverse Jacobian used for redundant manipulators.

## 4. Experimental Evaluation

We evaluate the performance of our proposed approach by deforming four objects with different shapes and material properties. The two shapes of the objects^2^ are described in Table 1 and the materials properties are shown in Table 2. The elastic behavior of isotropic materials can be represented by two parameters, such as the Young's modulus and the Poisson's ratio. One common way to obtain these parameters is to perform a compression test which consists on pushing down on an object while simultaneously recording the displacement^3^ of the object and the applied force.

**Table 1:** Geometric information of the test objects.

	**Dimensions (cm)**			**Mesh**	
	**Length**	**Width**	**Height**	**Nodes**	**Elements**
Block	6	40	40	360	1,079
Bar	6	6	50	207	536

**Table 2:** Material properties of the test objects.

		**Elasticity parameters**		
	**Material name**	**Mass density (*kg/m*^3^)**	**Young modulus (Pa)**	**Poisson ratio**
Hard	HR 45	45	18,500	0.15
Soft	Bultex 26	26	9,000	0.15

**Figure 5:**
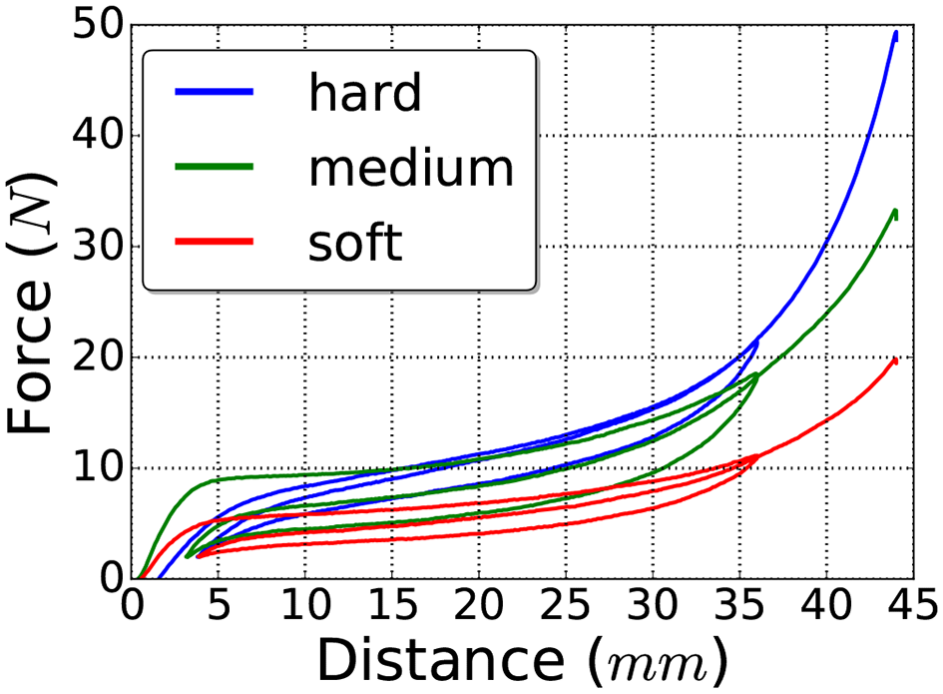
Displacement-force curve showing the hysteresis of the test materials.

We attempted to estimate the material properties of a cube object with three different materials: *hard* (HR 45), *medium* (Bultex 30), and *soft* (Bultex 26) (note that only the *hard* and *soft* materials were used in the experiments described in section 5). For the test, the object was placed inside of a press that can be programmed to move on a vertical axis until a desired height and is equipped with a force sensor. The press was set to first move down to approximately compress the object 36 *mm*, then to move upwards until the compression was around 4 *mm* and finally the press was moved down until 44 *mm* of compression were reached. The results of this test can be seen in Figure 5, where the compression is plotted against the measured force.

It can be seen from these figures, that the behavior of all three materials is not only non-linear but also presents hysteresis. Hence, modeling these materials as linear will result in inaccuracies. Nevertheless, we can use the test findings to obtain a range estimation of the Young's modulus for each material and then tune them individually until the simulation behavior better matches reality.

Two sets of experiments were conducted: one to measure the accuracy of the deformation sensing and the second one to assess the manipulation capabilities.

**Figure 6:**
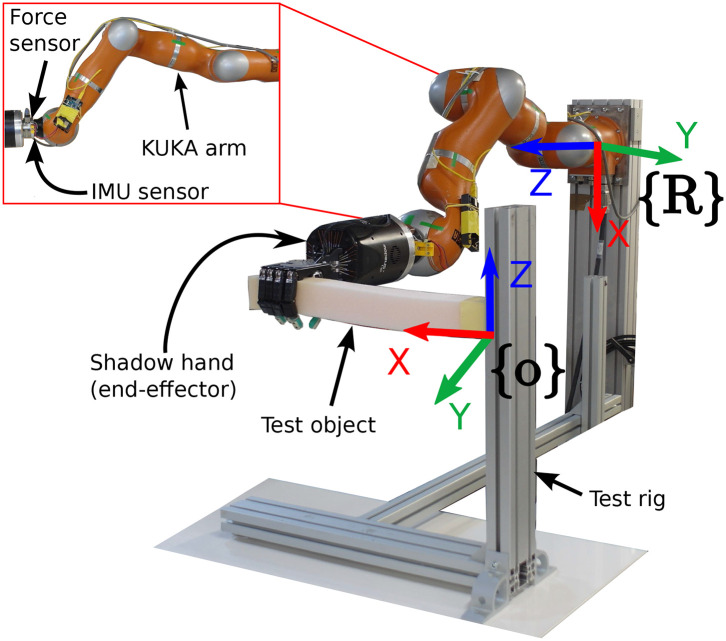
Experimental setup for a bar-like object.

The experimental setup used for both sets of experiments is shown in Figure 6, where the test rig ensures the contact points are consistent among the experiments. The seven degree of freedom robot manipulator KUKA LWR+4 arm (Bischoff et al., 2010) with the dexterous Shadow Dexterous Hand^4^ attached at its end were used to manipulate the objects. The inputs to the sensor model were obtained by an ATI Gamma^5^ force-torque sensor and an inertial measurement unit (IMU)^6^ sensor, both attached to the robot arm as shown in Figure 6. The test objects were fixed on a test rig, where the bar objects were fixed such that their long axis were parallel to the XY plane of the robot frame and the block objects were attached by their bottom side, as seen in Figures 6, 8.

The ANSYS^7^ software was used to generate the volumetric meshes of the four test objects and the Co-rotational Linear method from the Vega FEM library (Sin et al., 2013) was used to implement the deformation model. As for the force sensor model, we used the same network architecture described in Mohy el Dine et al. (2018) but trained the network with motions of the robot arm that were adequate for our experiments.

### 4.1. Deformation Sensing

**Figure 7:**
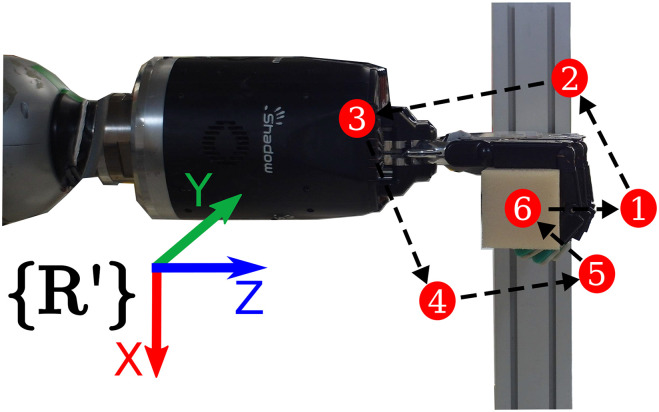
Example of the path to follow the six test poses by the bar objects during the sensing evaluation. The *R*′ denotes a reference frame having the same orientation as the robot base frame (see Figure 6) but a different translation in order to make it visible.

**Figure 8:**
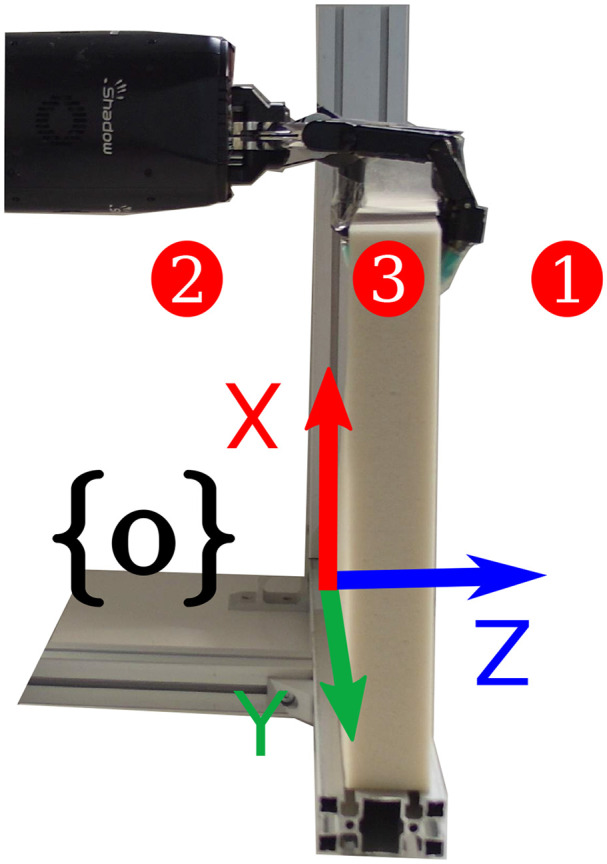
Test poses used for the block-like objects for the sensing evaluation.

To evaluate the sensing accuracy of our proposed pipeline we commanded a set of poses in the XZ plane of the robot frame, as shown in Figure 7. The bar objects had to reach the six different poses seen in Figure 7, where as the block objects were commanded to move to three poses along the Z axis (see Figure 8), since they were fixed on their bottom side. The error signal was computed as the distance per axis between the pose where the end-effector grasped the object (reference) and a pose on the mesh (measured). The measured pose was obtained by using the pose extraction method outlined in section 3.3, where the indices were chosen such that the pose coincided with the reference pose before the object was deformed.

**Figure 9:**
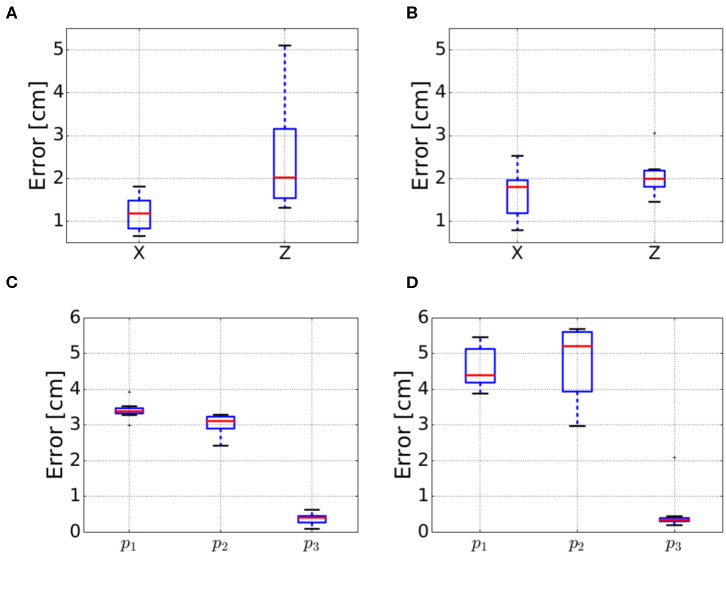
Errors for the deformation sensing evaluation for the four test objects. The errors for the bar objects refer to the average errors on the *X* and *Z* axes. For the block objects *p*_1_ refers to the front position, *p*_2_ to the back position and *p*_3_ is the original position after manipulating the object as shown in Figure 8. **(A)** Bar soft. **(B)** Bar hard. **(C)** Block soft. **(D)** Block hard.

For each object, seven trials were performed consisting in three and six target positions for the block and the bar objects, respectively. The results are summarized in Figure 9, where, the mean of the absolute error between the reference and measured positions was computed for each trial.

### 4.2. Manipulating Deformation

The performance of manipulating deformation was evaluated on the *bar soft* and *block hard* objects. For the bar object we set poses along the X and Z axis and the XZ plane as shown in Figure 7. As mentioned above, due to the block object being fixed at its bottom side, we only commanded poses along the Z axis as it can be seen in Figure 8. Similarly to the sensing evaluation, we defined the reference pose as the pose where the end-effector grasped the object and the measured pose was extracted using the pose extraction method as described above. Furthermore, we used a graphical user interface to set a target pose for the measured pose to reach.

Note that these control tasks could be solved by simply commanding a desired pose for the end-effector without regarding the mesh deformation. However, the purpose of this evaluation is to show that the shape of the object is estimated accurately enough to apply a controller.

## 5. Results and Experimental Insights

### 5.1. Sensing Evaluation

Before using the deformation sensing model in the control pipeline in Figure 3, it was evaluated quantitatively based on the experiments described in section 4.1 to draw ourselves and the reader an idea about the accuracy and precision of the model. The box plots for *X* and *Z* estimation of bar hard object are relatively small (< 2 cm) for 50% of the measurements, Figure 9A shows an accuracy of 1.6 cm in *X* and 2 cm in *Z*, however the precision is < 0.8 cm in *X* and < 0.3 cm in *Z*. Concerning the box plots of bar soft, for 50% of the measurements, they are < 2 cm as well in Figure 9B; the estimated position accuracy is 0.8 and 2 cm, respectively, in *X* and *Z* while the precision is < 0.4 cm in *X* and < 0.6 cm in *Z*. Concerning the blocks, the box plots are very small (< 2 mm) in Figures 9C,D. This reflects a 1 mm precision of the different measurements conducted for the both blocks. however, the accuracy is 3.8 cm along *Z* for the block hard and 2.25 cm for the block soft. The variances in the blocks position estimation measurements are smaller than the ones of the bars as the blocks are stiffer, hence they are more resistant to the small force reading errors. Regarding the different position accuracy measurements for the objects used, it is dependent on how good are the physical parameters in Table 2 applied to the mesh.

### 5.2. Soft Bar Experiments

**Figure 10:**
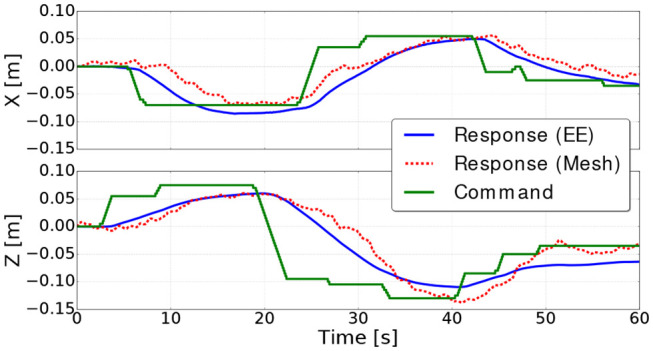
Command and responses of the mesh and the robot end effector (EE) along the *X* and *Z* axes for the bar soft object.

**Figure 11:**
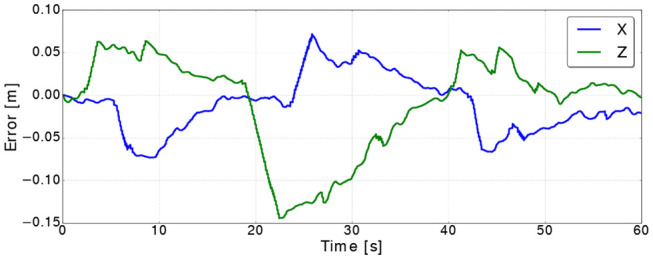
Control errors along the *X* and *Z* axes for the bar soft object.

The current pose estimation using the mesh is evaluated in the velocity control loop shown in the block diagram in Figure 3. Figure 10 shows the control position commands (green), along the *X* and *Z* axes, against the response positions extracted from the mesh (red) and the reference positions extracted using the robot kinematics (blue). The figures show that the controller is able to bring the mesh to the desired positions, along the *X* and *Z* axes, within a 2 cm error (the desired positions are reached at 19, 40, and 60 s) as shown in Figure 11. Moreover, in Figure 10, a clear delay can be seen between the command and the response of the mesh. This delay is mainly because of three reasons:

1. **Command:** Since the commanded signal is set by a GUI, the target poses for the controller result in a sharp slope.
2. **Elasticity parameters:** The deformation model is highly sensitive to the elasticity parameters, namely, the Young modulus and Poisson ration. Thus, an adequate identification is necessary.
3. **Sensor noise:** In order to obtain a smoother signal from the force sensors, a filter was applied but this introduced additional delay.

These reasons cause the controller to have a response similar to damped step response and takes around 20 s to converge to the commanded goal. Hence, the error increases the most when a command is received, then it converges to a value close zero (see Figure 11).

Concerning the small vibrations in the positions obtained from the mesh, they are mainly because of the small noise in the force readings. We note here that bar used in the tests is very soft (Table 2) that it can be deformed easily by small forces (< 2*N*), that's why the noises from residual non-contact forces and sensor noise have this much effect on the deformation model. For harder objects we expect the mesh to be less sensible to this noise.

### 5.3. Hard Block Experiments

**Figure 12:**
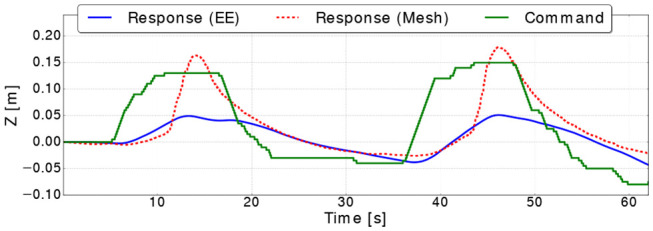
Command and responses of the mesh and the robot end effector (EE) along the *Z* axis for the block hard object.

**Figure 13:**
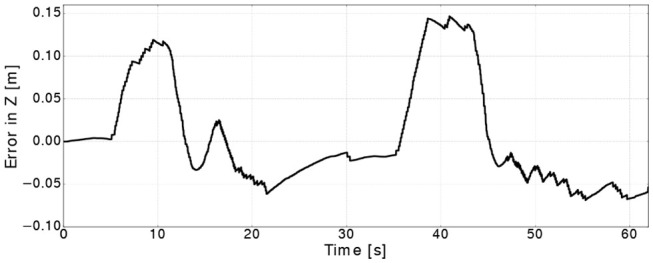
Control error along the *Z* for the block hard object.

The same controller shown in Figure 3 was used to move the block hard object to a different poses along the *Z* axes. Figure 12 shows the commanded position against the position extracted from the mesh, where the response of the controller shows a similar delay to the one in the bar experiment. However, the mesh estimation of the position overshoots as the target goal is far as almost twice as the one from the bar experiments. This is caused by the controller commanding the arm to move faster which in turn generates more residual inertial forces resulting in the mesh overshooting. It is important to note, that in these experiments we focus on measuring how accurate the controller can bring the mesh to a desired position without using vision sensors, rather than evaluating the controller's performance. Figure 13 shows the errors converge to less than 2 cm when the targets along the *Z* are reached (i.e., at 17, 35, and 45 s).

## 6. Conclusion and Future Work

We have presented a pipeline that is able to simultaneously estimate and manipulate deformable objects without the need of a vision system, which is widely required by other approaches. The proposed deformation sensing pipeline takes as input a contact force and an initial mesh with the physical characteristics of the object. An FEM simulation estimates the deformation of the mesh based on the input force and in order to control the mesh, a pose, extracted from a given set of nodes of the mesh, is driven by a robot manipulator to reach a desired pose. The approach was experimentally validated using a robotic arm with a dexterous hand as an end-effector and an attached force-torque sensors. The experimental validation showed promising results and precision to a certain limit.

The accuracy of the approach is limited due to several factors such as the control loop being closed on the pose extracted from the mesh which is subject to drift due to noise of the force-torque sensor readings. Additionally, the physical parameters of each object are not obtained in a straight forward manner, thus affecting the estimation between the deformation of the real object and the estimation by the model. In future work, we will focus on incorporating vision to correct the errors in the estimation of the deformation, as well as investigating the performance of different controllers for deformation control found in the literature (e.g., Navarro-Alarcon et al., 2016; Hu et al., 2018). Furthermore, this approach can be extended to multi-robot systems, such that each robot controls a pose on the mesh and thus increasing the shape control capabilities.

## Data Availability Statement

All datasets generated for this study are included in the article/supplementary material.

## Author Contributions

All the authors participated in the development and validation of the research work presented in this paper.

## Conflict of Interest

The authors declare that the research was conducted in the absence of any commercial or financial relationships that could be construed as a potential conflict of interest.

## Footnotes

^1^An implementation of the code is available at https://github.com/jsanch2s/uca_deformation_sensing.

^2^The objects were bought from the following vendor: http://www.moussesurmesure.com/.

^3^Here, displacement refers to the compression of the object, i.e., the object is fixed at one side while the opposite side is moved toward the fixed side.

^4^https://www.shadowrobot.com/products/dexterous-hand/.

^5^http://www.ati-ia.com/products/ft/ft_models.aspx?id=Gamma.

^6^We used the Adafruit (L3GD20H + LSM303) sensors: https://www.adafruit.com/product/1714.

^7^https://www.ansys.com/.
